# Effect of Fermentation Time on the Physicochemical Properties and Phenolic Composition of Malted Finger Millet Beverages

**DOI:** 10.1002/fsn3.4659

**Published:** 2025-03-10

**Authors:** Henry Okwudili Udeh, Kwaku Gyebi Duodu, Afam Israel Obiefuna Jideani

**Affiliations:** ^1^ Department of Food Science and Technology, Faculty of Science, Engineering and Agriculture University of Venda Thohoyandou South Africa; ^2^ Department of Applied Microbiology and Brewing, Faculty of Natural and Applied Sciences State University of Medical and Applied Sciences Nsukka Enugu State Nigeria; ^3^ Department of Consumer and Food Sciences, Faculty of Natural and Agricultural Sciences University of Pretoria Pretoria South Africa; ^4^ Special Interest Group Post Harvest Handling ISEKI‐Food Association Vienna Austria

**Keywords:** Antioxidant activity, *Eleusine coracana*, fermentation, phenolic compounds, physicochemical properties

## Abstract

Food security is still a significant problem, particularly in less developed nations. Orphan crops, such as finger millet (FM), are essential for meeting dietary and nutritional needs as well as providing a means of livelihood for economies with limited resources in Asia and Africa. Comparing two varieties of FM malt beverages using sorghum grain as an external reference, the research examined the impact of fermentation time on the physicochemical characteristics and phenolic composition of the FM malt beverages. The FM grains were fermented using *Lactobacillus fermentum* and the grain microflora. The beverages' pH decreased in a time‐dependent manner throughout fermentation, and their sugar content increased accordingly. A reduction in the beverages' viscosities for both *L. fermentum* and the grain microflora was observed. The amount of citric acid in the beverages decreased as fermentation progressed, especially for *L. fermentum* . Catechin, epicatechin, and protocatechuic acid were the phenolics identified in the FM beverages. An increase in fermentation time was correlated with a decrease in the beverages' total phenolic content. At 96 h of fermentation, the beverages' DPPH, ABTS, and FRAP radical scavenging activities were significantly (*p* < 0.05) reduced for both *L. fermentum* and the grain microflora. The 24‐h fermented beverages maintained higher amounts of total polyphenols and antioxidant effects. The findings indicate that FM may be utilized as a functional grain to produce nonalcoholic beverages with health benefits.

## Graphical abstract

The study investigated the effect of fermentation time on the physicochemical properties and phenolic composition of two cultivars of finger millet (FM) malt beverages, compared to sorghum. The 24‐h fermented FM malt beverages retained a higher amount of total phenolics and had higher antioxidant activity. The study shows that FM could be used as a functional grain in the production of nonalcoholic beverages with health benefits.

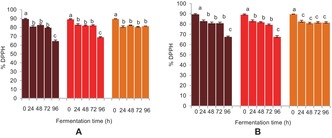

## 1 Introduction

Finger millet (FM), *Eleusine coracana* (L.) Gaertn is categorized as an excellent food crop by the US National Academies, ranking it among the major nutrient‐dense cereals (NRC 1996; Udeh, Duodu, and Jideani 2018; Mishra et al. 2024). FM accounts for 75% of total caloric intake, making it a key food source in the emerging economies of the African and Asian continents (Xiang et al. 2019; Maharajan et al. 2021). It is a tiny, seeded crop that is a member of the subfamily *Chloridodeae* and the grass family *Poaceae*, which has its origin in Ethiopia (Sood et al. 2016). FM grain is available in three distinct varieties: light brown, brown, and white. Varietal distinction and use are based mostly on grain color (Kumar et al. 2016; Ramashia et al. 2019). In Southern Africa, conventional opaque beer is brewed using the brown variety, whereas the light a brown varieties are used to make porridge. The white cultivars were primarily produced for the baking sector (Sood, Kant, and Pattnayak 2017). Notwithstanding its nutritional, functional, and health‐promoting properties, FM has been underutilized, and there is little innovation in FM beverages in contrast to other cereals such as maize and sorghum. For example, the use of sorghum (as a whole ingredient or adjunct) in the manufacture of a variety of beverages and other food items is well documented (Chelule, Mokoena, and Gqaleni 2010; Khalid et al. 2022; Adu et al. 2024; Mashau, Muluvhu, and Ramashia 2024), but similar information on FM is comparatively scarce or not available, particularly in Southern Africa.

Important nutrients such as dietary fiber, minerals, vitamins, and phytochemicals with several health benefits can be found in FM. It is a low‐glycemic, gluten‐free cereal crop that has excellent malting properties and makes a nutritious substitute for gluten‐free and diabetic diets (Aljobair 2022; Patil, Singh, and Patel 2023; Kalsi et al. 2023; Joseph et al. 2024; Murungweni et al. 2024). FM has a higher micronutrient density than rice and wheat and a notably higher concentration of phenolic compounds, calcium, and other important minerals. The FM phenolics occur in free, conjugated, and bound forms. Protocatechuic and ferulic acids constitute the major free and bound fractions of FM, whereas catechin and epicatechin are the predominant flavonoids (Xiang et al. 2019; Jacob et al. 2024). There has been extensive reporting on FM phenolic compounds (Shahidi and Chandrasekara 2013; Devi et al. 2014; Udeh, Duodu, and Jideani 2017). Major antioxidant, antidiabetic, anticarcinogenic, antihypertensive, antimicrobial, and antiaging activities are exhibited by FM phenolic components, such as quercetin, catechin, epicatechin protocatechuic, and ferulic acids, among others, and they efficiently reduce cataract development (Bae et al. 2020; Maharajan et al. 2021; Ibrahem and Sayed 2023; Jeong et al. 2024; Jacob et al. 2024; Mahanta et al. 2024; Widowati et al. 2024).

For food and beverage items, phenolic compounds are key because they enhance their sensory and nutritional qualities. Food products' quality is closely linked with these compounds, and as such, their analysis is of significant interest. Sripriya, Anthony, and Chandra (1997) documented a rise in the total polyphenols of FM fermented with the naturally occurring microorganisms in the grain, whereas Rani and Anthony (2014) indicated that the grain's total phenolics (TP) decreased in a time‐dependent manner throughout a 48‐h fermentation. However, the impact of fermentation time on the total phenols and antioxidant activities of the fermented FM was inconclusive and therefore requires further studies on the processing, physicochemical, and phenolic profiling of FM beverages. Data on the influence of fermentation time on the phenolics and antioxidant activities of FM malt beverages are scarce. Thus, using a pure culture of lactic acid‐fermenting bacteria and the endogenous FM grain microflora, this study examines the physicochemical and health‐promoting characteristics of beverages made from two varieties of FM malt flour.

## 2 Materials and Methods

The red‐colored, nontannin sorghum (type I) and the local varieties of FM, brown (BFM), and dark brown (DBFM) were bought from a retail store in Thohoyandou, Limpopo Province, South Africa. The *Lactobacillus fermentum* ATCC 9338 pure culture was acquired from Anatech Instruments, located in South Africa. MRS nutritional broth and de Man, Rogosa, and Sharpe (MRS) agar were acquired from Lab M Limited in the United Kingdom. The Folin–Ciocalteu reagent, gallic acid, quercetin, 2, 2‐diphenyl‐1‐picrylhydrazyl, 2, 2‐azinobis‐3‐ethylbenzthiazoline‐6‐sulfonic acid, and Trolox standards were procured from Rochelle Chemicals and Laboratory Equipment in Johannesburg, South Africa. The solvents used for ultraperformance liquid chromatography‐mass spectrometry were of chromatographic grade.

### 2.1 Malting of the FM and Sorghum Grains

The process was carried out using Chethan, Sreerama, and Malleshi (2008) methodology. Since most nonalcoholic beverages in South African retail establishments are manufactured from sorghum grain, sorghum was utilized as an external reference to produce similar nonalcoholic beverages from FM malt. In a growth chamber, 100 g portions of the rinsed cereals were immersed in water at 25°C for 24 h. After the soaking period, the water was drained, and the cereal grains were allowed to germinate by spreading them out on a clean cheesecloth with distilled water sprinkled on them every 24 h. The germinated cereal grains were withdrawn at 24 h intervals for 96 h. Following an 8‐h kilning at 50°C, the sprouted grains were ground into flour and kept in airtight polythene bags at −20°C.

### 2.2 Fermentation of the FM and Sorghum Malt Flours

**FIGURE 1:**
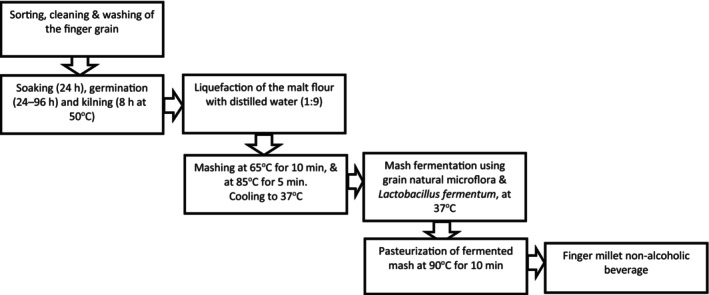
Flow diagram for the production of fermented finger millet and sorghum malt nonalcoholic beverage. Adapted from Chelule, Mokoena, and Gqaleni (2010).

The fermentation process was performed by adapting the Chelule, Mokoena, and Gqaleni (2010) procedure (Figure 1). The flour was made by adding and mixing the flour with water in a ratio of 1:9 w/v in a 250‐mL volumetric flask. Starting at 25°C, with the aid of a hot water bath, the mash temperature was progressively increased and kept at 65°C for 10 min. A further increase in the mash temperature was done and maintained at 85°C for 5 min. The *L. fermentum* inoculum was prepared using the Gao, Qiao, and Lu (2009) method. Following a 48‐h growth period at 37°C, the cells were collected by centrifugation (4470 g, 15 min) and washed twice with ozonated water. The grain microbes were cultured by adding 3 g of unmalted flour to ozonated water, which was allowed to grow at 37°C for 20 min before use. Fermentation was performed with the grain microflora and *L. fermentum* independently for 96 h at 37°C in an incubator. The fermented mash was taken out every 24 h and pasteurized for 10 min at 90°C.

### 2.3 Viscosity, pH, and Sugar Content of the Fermented FM and Sorghum Malt Beverages

Using a Brookfield viscometer set to spindle 7, speed 100 rpm, and temperature 25°C, the viscosity of the beverages was measured. The pH of the beverages was measured with a digital pH meter at room temperature. An automatic refractometer was used to determine the sugar content of the beverages following the manufacturer's instructions.

### 2.4 Preparation of Phenolic Extracts From the Fermented FM and Sorghum Malt Beverages

The polyphenolic extracts were prepared by heating 5 g of the beverage sample with 20 mL of 1% HCl‐methanol at 60°C for 2 h in an airtight 50 mL test tube (Chethan and Malleshi 2007). Following a 20‐min centrifugation at 4470 *g*, the supernatants were decanted, which were then analyzed for the phenolic profile and antioxidant activities of the beverages.

### 2.5 Phenolic Compounds and Citric Acid of the Fermented FM and Sorghum Malt Beverages

Waters Synapt G2 quadrupole time‐of‐flight mass spectrometer system (MS) [Milford, MA, USA] was used to analyze the phenolic components and citric acid content of the beverage extracts using the methodology of Udeh (2018). The citric acid content of the beverages was deemed necessary to include in the study, as a significant amount of the organic acid was detected in the samples during analysis of the phenolic compounds. The system was equipped with a photodiode array (PDA) detector and ultraperformance liquid chromatography (LC). A Waters BEH C18, 2.1 × 100 mm column with 1.7‐μm particles was used to achieve separation. Solvents A and B, which were acetonitrile and 0.1% formic acid, respectively, were used to apply a gradient. In a linear pattern, the gradient changed from 100% solvent A for 1 min to 28% solvent B over 22 min. After a 50‐s transition to 40% B, a 1.5 min 100% B wash step, and a 4 min re‐equilibration period, it changed back to initial conditions. The injection volume was 2 μL, the column was maintained at 55°C, and the flow rate was 0.3 mL/min. Two scans were conducted: one with a high collision energy from *m*/*z* 40 to 1500 and the other with a low collision energy (6 V) from *m*/*z* 150 to 1500. The data were collected in MS^E^ mode. A collision energy ramp of 30–60 V was used to perform the high collision energy scan. The scan range for the photodiode array detector was tuned to 220–600 nm. For maximum sensitivity, the mass spectrometer was optimized using nitrogen desolvation gas at 650 L/h, a desolvation temperature of 275°C, and a cone voltage of 15 V. The device was run in the negative mode using an electrospray ionization probe. Accurate mass estimations were achieved by infusing leucine encephalin as a lock mass in the background and using sodium formate for calibration. By comparing the retention time and spectra of each peak with known standards under the same conditions, individual peaks and phenolic compounds were confirmed.

### 2.6 Total Phenolic Content of the Fermented FM and Sorghum Malt Beverages

The Singleton, Orthofer, and Lamule‐Raentos (1995) methods were used to calculate the beverages' total phenolic content. Briefly, a 0.1 mL extract of the beverage was added to 5 mL of distilled water in a 50‐mL volumetric flask. Folin–Ciocalteu's reagent (2.5 mL) and 7.5 mL of 15% sodium carbonate were added and mixed well, made up to 50 mL, and allowed to react for 30 min. The mixture's absorbance was measured using a 96‐well microplate spectrophotometer at 760 nm. The result was expressed as milligram gallic acid equivalent per gram dry basis.

### 2.7 Total Flavonoids of the Fermented FM and Sorghum Malt Beverages

The total flavonoid (TF) content of the beverages was determined using the procedure of Zhishen, Mengcheng, and Jianming (1999) and Udeh (2018). Accurately, 0.1 mL of the beverage extract and 4.9 mL of distilled water were mixed, to which 0.3 mL of NaNO_2_ (5% w/v) was added. After 5 min, AlCl_3_ (0.3 mL, 10% w/v) was added, followed by 2 mL of 1 M NaOH at 6 min. Distilled water was then added to bring the volume up to 10 mL. After vortexing the mixture, the absorbance at 510 nm was measured. The result was given as milligram quercetin (QE) per gram dry basis.

### 2.8 2, 2‐Diphenyl‐1‐Picrylhydrazyl (DPPH) Radical Scavenging Activity of the Fermented FM and Sorghum Malt Beverages

De Ancos et al. (2002) and Udeh (2018) methodologies were used to ascertain the DPPH radical scavenging activity of the beverages. Exactly 10 μL of the extract was added to 90 μL of distilled water and 3.9 mL of a methanolic 0.1 mM DPPH solution. The mixture was vortexed and left in the dark for 30 min to react before the absorbance was measured at 515 nm. The outcome was given as the percentage inhibition of the DPPH radical, which was determined using the following equation:

$$\%\mathrm{Inb}=\left(\mathrm{Ab}\;\text{control}–\mathrm{Ab}\;\text{sample}/\mathrm{Ab}\;\text{control}\right)\times 100.$$

Ab control, absorbance of DPPH solution without beverage extract. %Inb, percentage inhibition of DPPH.

### 2.9 2, 2‐Azinobis‐3‐Ethylbenzthiazoline‐6‐Sulfonic Acid (ABTS) Radical Scavenging Activity of the Fermented FM and Sorghum Malt Beverages

With slight modification, the beverage assay was determined using the technique described by Thaipong et al. (2006) and Arnao, Cano, and Acosta (2001). To create ABTS^+^ radicals, equal volumes (10 mL) of potassium persulfate (2.6 mM) and 7.4 mM ABTS (made in distilled water) were combined and left for 12 h in the dark at room temperature. To achieve an absorbance of 0.098 at 734 nm, the solution was mixed with 1 mL of ABTS and 60 mL of phosphate buffer (0.1 M NaOH, pH 7.4). After allowing the beverage extract (150 μL) to react with 2850 μL of the ABTS^+^ radical solution for 5 min, the absorbance was read at 734 nm. The standard curve was obtained by mixing Trolox and phosphate buffer at pH 7.4 and reacting it with 2850 μL of the ABTS radicals for 5 min. The outcomes were presented as μM Trolox equivalent per gram.

### 2.10 Ferric‐Reducing Antioxidant Power (FRAP) of the Fermented FM and Sorghum Malt Beverages

The FRAP was ascertained following the methodology of Oyaizu (1986) and Udeh (2018). Accurately, 100 μL of the supernatant was measured, and methanol was used to increase the volume to 1 mL. Precisely, 2.5 mL of potassium ferricyanide (1%) and 2.5 mL of 0.2 M phosphate buffer (pH 6.6) were added and thoroughly mixed. The combination was allowed to react for 20 min at 50°C in a hot water bath. Following this period, 2.5 mL of 10% (w/v) trichloroacetic acid was added and then centrifuged (4470 g, 20 min). The supernatant (2.5 mL) was collected and mixed with distilled water (2.5 mL) and 0.5 mL of 0.1% (w/v) ferric chloride. The absorbance was read at 700 nm. A higher absorbance value corresponds to a higher reducing power.

### 2.11 Statistical Analysis

The generated data were analyzed using SPSS for Windows version 24. The differences in mean values were determined using a one‐way analysis of variance, followed by Duncan's multiple comparison test. The significance of the correlation was ascertained with the Pearson correlation test, and the significance levels of the mean values were determined at *p < 0.05 and **p < 0.01.

## 3 Results

### 3.1 Viscosity, pH, and Sugar Content of the Fermented FM and Sorghum Malt Beverages

**FIGURE 2:**
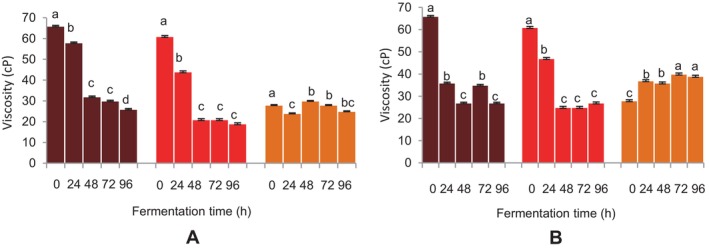
Effect of fermentation time on the viscosity of finger millet and sorghum malt beverages: 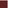 dark brown finger millet; 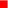 brown finger millet; 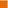 sorghum. (A) Fermentation with *Lactobacillus fermentum* . (B) Fermentation with grain microbial flora. Bars are mean values of triplicate determinations ± standard error. For each fermentation period, mean values with different letters are significant at *p* < 0.05.

A fermentation‐time‐dependent decrease in viscosity was observed for the FM beverages fermented with *L. fermentum* (Figure 2A). A similar decrease was noted for the sorghum beverage after 24 h of fermentation, which shortly increased and remained the same up to 72 h (Figure 2A). A drop in the viscosities of the FM beverages was noted up to 48 h, which remained unchanged up until 96 h for BFM beverages fermented with the grain microflora (Figure 2B). Conversely, the sorghum beverage's viscosity showed a steady increase up to 96 h of fermentation (Figure 2B).

**FIGURE 3:**
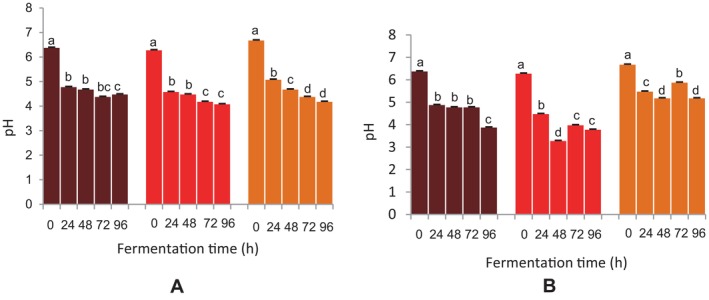
Effect of fermentation time on pH of finger millet and sorghum malt beverages. 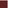 Dark brown finger millet; 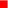 Brown finger millet; and 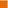 sorghum. (A) Fermentation with *Lactobacillus fermentum* . (B) Fermentation with grain microbial flora. Bars are mean values of triplicate determinations ± standard error. For each fermentation period, mean values with different letters are significant at *p* < 0.05.

Following 96 h of fermentation, a drop in pH was observed in the FM and sorghum beverages fermented using *L. fermentum* (Figure 3A). A comparable decrease in the pH of the beverages was also observed with the grain microflora. A relatively substantial downturn in the pH of the DBFM beverage was seen at 96 h using the grain microflora (Figure 3B). Similar observations were noted for the BFM and sorghum beverages, except at 72 h, where an increase in pH was recorded.

**FIGURE 4:**
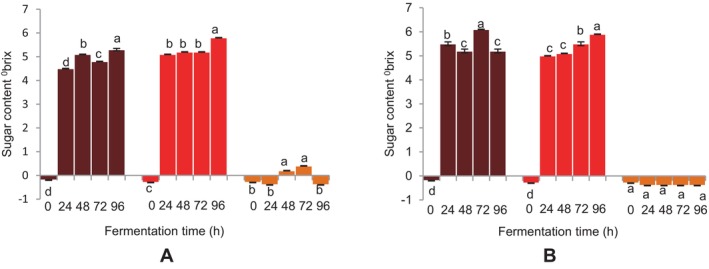
Effect of fermentation time on sugar content of finger millet and sorghum malt beverages: 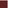 dark brown finger millet; 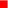 brown finger millet; and 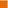 sorghum. (A) Fermentation with *Lactobacillus fermentum* . (B) Fermentation with grain microbial flora. Bars are mean values of triplicate determinations ± standard error. For each fermentation period, mean values with different letters are significant at *p* < 0.05.

With the BFM beverage fermented with *L. fermentum* , an increase in the ^o^brix or sucrose content was observed with a corresponding increase in fermentation time (Figure 4A). The DBFM beverage also showed a comparable increase in sugar content. A rise in sugar content was seen up to 96 h into the BFM beverage's fermentation with the grain microflora; however, this increase was only seen at 72 h into the DBFM beverage's fermentation (Figure 4B). There were no detectable sugars in the sorghum beverage fermented with the grain microflora; however, an increase in the sugar content was noted for the *L. fermentum* at 48 and 72 h for the same (Figure 4A).

### 3.2 Phenolic Composition of the Fermented FM and Sorghum Malt Beverages

Table 1 describes the mass spectra features and retention times of the phenolic components found in malt beverages. Benzoic acid derivatives, flavan‐3‐ols or flavanols, flavononols, and flavonols were the groups of phenolic compounds found in the beverages. Respectively, the phenolic compounds identified in each phenolic group in the beverages are as follows: benzoic acid derivative: protocatechuic acid; flavanols: catechin and epicatechin; flavononol: taxifolin; and flavonols: kaempferol.

**TABLE 1:** Retention time and mass spectral characteristics of phenolic compounds identified in fermented finger millet and sorghum malt beverages.

*t*_R_ (min)	[M−H]^−^ (*m*/*z*)	MS/MS fragments (intensity, %)	Identified compounds
*Benzoic acid derivatives*			
8.70	153	153 (100)	Protocatechuic acid
*Flavanols*			
12.03	289	289 (100), 245 (5)	Catechin
14.08	289	289 (100)	Epicatechin
*Flavononol*			
15.40	303	303 (100), 285 (49)	Taxifolin
*Flavonols*			
10.23	285	285 (100)	Kaempferol

Abbreviations: [M−H]^−^, negative ion mode; MS, mass spectra; *t*_R_, retention time.

### 3.3 Concentration of Phenolic Compounds in FM and Sorghum Malt Beverages

A decrease in the total concentration of phenolics was observed during the fermentation of the beverages (Table 2). Fermentation for 24 h reduced the catechin and epicatechin concentrations in the DBFM beverage for both microbial sources. A similar reduction in the same was noted in the BFM beverage. After 48 h of fermentation, the BFM beverage fermented with *L. fermentum* showed no substantial reduction in the amount of protocatechuic acid. Fermentation up to 96 h with the grain microflora showed no considerable difference in the amounts of epicatechin and catechin in the sorghum beverage. For the sorghum beverage fermented with *L. fermentum* , no substantial differences in catechin and epicatechin concentrations were recorded up until 72 h.

**TABLE 2:** Effect of fermentation time on concentration (μg/g) of phenolic compounds in finger millet and sorghum malt beverages.

Beverage samples/compounds	Fermentation time (h)									
	0	24	48	72	96	0	24	48	72	96
	Grain natural microflora					*Lactobacillus fermentum*				
*Dark brown finger millet*										
PA	0.90 ± 0.14^a^	0.75 ± 0.07^a^	0.70 ± 0.14^a^	0.30 ± 0.00^b^	0.40 ± 0.14^b^	0.90 ± 0.14^a^	0.70 ± 0.14^abc^	0.75 ± 0.21^bc^	0.50 ± 0.00^ab^	0.55 ± 0.28^c^
CA	6.25 ± 0.28^a^	5.10 ± 0.14^b^	6.05 ± 0.21^a^	2.50 ± 0.64^c^	1.85 ± 0.85^c^	6.25 ± 0.28^a^	4.10 ± 0.00^b^	2.85 ± 0.64^c^	1.45 ± 0.35^d^	1.40 ± 0.14^d^
ECA	1.25 ± 1.13^a^	0.90 ± 0.00^b^	1.00 ± 0.00^ab^	0.45 ± 1.06^c^	0.30 ± 0.00^c^	1.25 ± 1.13^a^	0.65 ± 0.21^b^	0.40 ± 0.14^cb^	0.25 ± 0.07^cb^	0.20 ± 0.00^c^
Total	8.40 ± 1.55	6.75 ± 0.21	7.96 ± 0.35	2.95 ± 1.70	2.55 ± 0.99	8.40 ± 1.55	5.45 ± 0.35	4.00 ± 0.99	2.20 ± 0.42	2.15 ± 0.42
*Brown finger millet*										
PA	1.70 ± 0.28^a^	0.70 ± 0.00^b^	0.50 ± 0.14^c^	0.95 ± 0.07^bd^	1.00 ± 0.00^bd^	1.70 ± 0.28^a^	1.25 ± 0.21^a^	0.95 ± 0.21^ab^	1.40 ± 0.14^b^	0.80 ± 0.07^b^
CA	7.10 ± 0.35^a^	3.50 ± 0.78^b^	2.85 ± 0.28^b^	6.00 ± 0.42^a^	5.75 ± 0.21^a^	7.10 ± 0.35^a^	4.40 ± 0.14^b^	3.30 ± 0.42^c^	3.65 ± 0.07^cb^	2.60 ± 0.14^cd^
ECA	1.45 ± 0.99^a^	0.55 ± 0.78^b^	0.55 ± 0.99^b^	1.00 ± 0.00^ab^	0.90 ± 0.84^b^	1.45 ± 0.99^a^	0.90 ± 0.00^b^	0.55 ± 0.07^c^	0.70 ± 0.00^bc^	0.50 ± 0.00^c^
Total	10.25 ± 1.62	4.75 ± 1.56	3.90 ± 1.41	7.95 ± 0.49	7.65 ± 1.05	10.25 ± 1.62	6.55 ± 0.35	4.80 ± 0.70	5.75 ± 0.21	3.90 ± 0.21
*Sorghum grain*										
PA	2.35 ± 0.49^a^	1.75 ± 0.21^ab^	1.30 ± 0.14^bc^	1.25 ± 0.07^bc^	1.40 ± 0.21^bc^	2.35 ± 0.49^a^	1.55 ± 0.14^b^	1.50 ± 0.28^b^	1.25 ± 0.35^b^	1.40 ± 0.63^b^
CA	5.35 ± 1.34^a^	5.35 ± 1.13^a^	5.10 ± 1.48^a^	4.70 ± 2.05^a^	5.65 ± 1.91^a^	5.35 ± 1.34^a^	2.80 ± 0.28^b^	4.15 ± 0.07^ba^	4.35 ± 1.20^ba^	3.60 ± 0.71^bc^
ECA	0.15 ± 1.06^a^	0.15 ± 0.99^a^	0.20 ± 0.00^a^	0.25 ± 1.13^a^	0.25 ± 1.20^a^	0.15 ± 1.06^a^	0.20 ± 0.00^a^	0.10 ± 0.00^a^	0.15 ± 0.07^a^	0.10 ± 0.00^a^
TX	1.85 ± 0.85^a^	2.00 ± 0.00^a^	1.65 ± 0.78^b^	1.75 ± 0.85^ba^	1.75 ± 1.06^ba^	1.85 ± 0.85^a^	1.00 ± 0.42^b^	0.80 ± 0.00^b^	1.15 ± 0.07^b^	1.00 ± 0.14^b^
KF	1.15 ± 0.78^a^	1.50 ± 0.92^ab^	1.65 ± 0.71^b^	1.35 ± 0.92^ab^	1.75 ± 1.13^b^	1.15 ± 0.78^a^	Nd	Nd	1.20 ± 0.14^a^	1.05 ± 0.21^a^
Total	10.85 ± 4.52	10.75 ± 3.25	9.90 ± 3.11	9.30 ± 5.02	10.80 ± 5.51	10.85 ± 4.52	5.55 ± 0.84	6.55 ± 0.35	8.10 ± 1.83	7.15 ± 1.69

*Note:* Values given are mean values of duplicate experiments; mean values in a row with different superscripts are significantly different at *p* < 0.05.

Abbreviations: CA, (+)‐catechin; ECA, (−)‐epicatechin; KF, kaempferol; Nd, not detected; PA, protocatechuic acid; TX, taxifolin.

At 48 h of fermentation with the grain microflora, there was a considerable increase in the phenolic components of the BFM beverage (Table 2). Conversely, at 48 h of fermentation, reductions were observed for both microbial sources in the DBFM beverage. The phenolic compounds in the sorghum beverage decreased in tandem with fermentation time for microbial sources, except taxifolin and kaempferol, which showed increases, especially at 96 h. Notwithstanding the decreases in phenolic content observed during the fermentation durations, 24‐h fermentation retained high total phenolic concentrations for the FM beverages.

### 3.4 Citric Acid Content of the Fermented FM and Sorghum Malt Beverages

**FIGURE 5:**
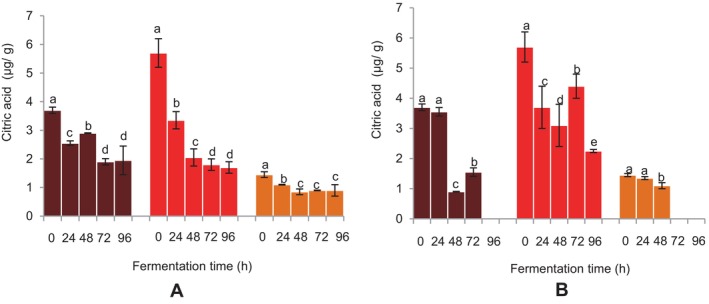
Effect of fermentation time on the citric acid content of the finger millet and sorghum malt beverages: 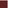 dark brown finger millet; 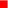 brown finger millet; and 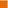 sorghum. (A) Fermentation with *Lactobacillus fermentum* . (B) Fermentation with grain microbial flora. Bars are mean values of triplicate determinations ± standard error. For each fermentation period, mean values with different letters are significant at *p* < 0.05.

At 72 h of fermentation using *L. fermentum* , there was a considerable drop in the BFM beverage's citric acid level, which remained unchanged up until 96 h (Figure 5A). A similar observation was noted for the DBFM beverage fermented with *L. fermentum* . During fermentation with the grain microflora, a decrease in the citric acid level of the BFM beverage was noted, except at 72 h of fermentation, where a substantial increase in the same was recorded (Figure 5B). A similar observation was made in the citric acid content of the DBFM beverage fermented with the grain microflora, which was undetected at 96 h of fermentation. A fermentation‐time‐dependent decline in the citric acid level of the sorghum beverage was seen up to 48 h with *L. fermentum* , which remained unchanged until 96 h (Figure 5A). At 48 h into the fermentation process with the grain microflora, there was a corresponding drop in the sorghum beverage's citric acid concentration; after that, it was undetected (Figure 5B).

### 3.5 TP and Total Flavonoids of the Fermented FM and Sorghum Malt Beverages

**FIGURE 6:**
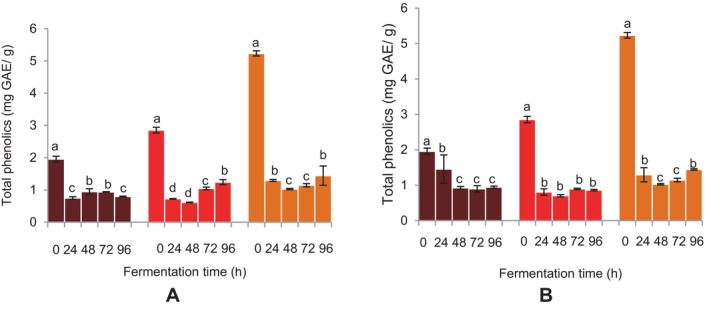
Effect of fermentation time on total phenolic content of finger millet and sorghum malt beverages: 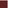 dark brown finger millet; 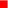 brown finger millet; and 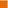 sorghum. (A) Fermentation with *Lactobacillus fermentum* . (B) Fermentation with grain microbial flora. Bars are mean values of triplicate determinations ± standard error. For each fermentation period, mean values with different letters are significant at *p* < 0.05.

The TP of the beverages showed a significant decrease at 24 h of fermentation for both microbial sources (Figure 6A,B). For BFM and sorghum beverages fermented with *L. fermentum* , a further drop in TP was seen after 48 h, and this increase remained constant until 96 h (Figure 6A). In contrast, an increase in TP was noted at 48 and 72 h for the DBFM beverages fermented with *L. fermentum* , followed by a reduction at 96 h. A drop in TP was recorded at 24 and 48 h of fermentation for the DBFM beverages fermented with the grain microflora, and this decline persisted until 96 h. A comparable observation was seen at 24 h for BFM beverages using the grain microflora, which remained unchanged up until 96 h. A significant decrease in TP was observed at 24 and 48 h for the sorghum beverage fermented with the grain microflora, after which there was an increase up to 96 h (Figure 6B).

**FIGURE 7:**
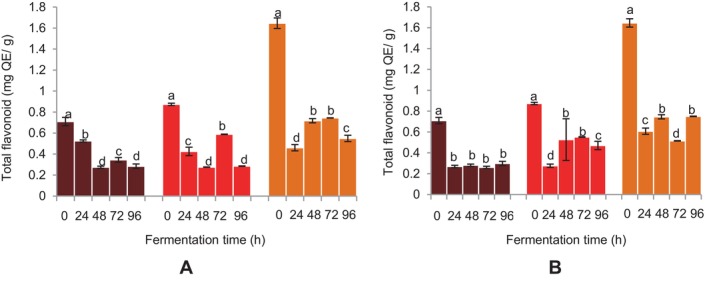
Effect of fermentation time on total flavonoids of finger millet and sorghum malt beverages: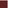 dark brown finger millet; 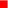 brown finger millet; and 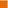 sorghum. (A) Fermentation with *Lactobacillus fermentum* . (B) Fermentation with grain microbial flora. Bars are mean values of triplicate determinations ± standard error. For each fermentation period, mean values with different letters are significant at *p* < 0.05.

A considerable decrease in TFs of the beverages was observed at 24 h of fermentation with *L. fermentum* (Figure 7A). Using *L. fermentum* , a rise in TF of the FM beverages was seen at 72 h, which subsequently decreased at 96 h. A parallel increase in TF was noted for the sorghum beverage at 48 and 72 h, which later decreased after 96 h of fermentation (Figure 7A). No substantial (*p* > 0.05) changes in TF were recorded for DBFM beverages fermented with the grain microflora, except for the initial decline at 24 h (Figure 7B). A similar decrease in the TF was noted for BFM and sorghum beverages after 24 h, followed by an increase at 48 and 72 h for BFM beverages that declined at 96 h of fermentation using the grain microflora (Figure 7B). The sorghum beverage showed similar increases in TF at 48 and 96 h, with a decrease in TF noted at 72 h of fermentation using the grain microflora (Figure 7B).

### 3.6 DPPH and ABTS Radical Scavenging Activities and FRAP of Fermented FM and Sorghum Malt Beverages

**FIGURE 8:**
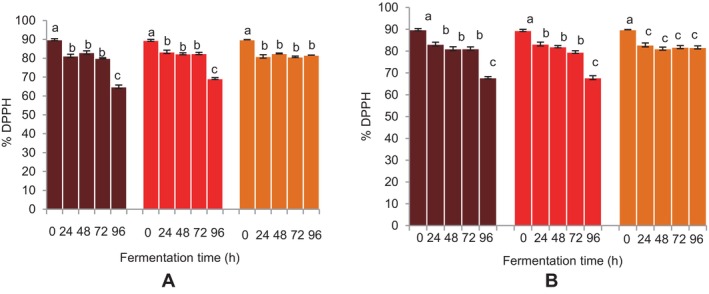
Effect of fermentation time on DPPH (2, 2′‐diphenyl‐1‐picrylhydrazyl) radical scavenging activity of finger millet and sorghum malt beverages: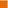 dark brown finger millet; 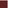 brown finger millet; and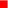 sorghum. (A) Fermentation with *Lactobacillus fermentum* . (B) Fermentation with grain microbial flora. Bars are mean values of triplicate determinations ± standard error. For each fermentation period, mean values with different letters are significant at *p* < 0.05.

The beverages' capacity to scavenge DPPH radicals was initially reduced as a result of fermentation using both microbial sources (Figure 8A,B). No substantial (*p* > 0.05) changes in the DPPH radical scavenging activities of the FM beverages were noted after 24 h up until 72 h, especially with *L. fermentum* . The DPPH radical scavenging activities of the FM beverages fermented with both microbial sources were considerably reduced after 96 h of fermentation. No significant changes in the DPPH radical scavenging activities of the sorghum beverage were noted after 24 h of fermentation for both microbial sources (Figure 8A,B).

**FIGURE 9:**
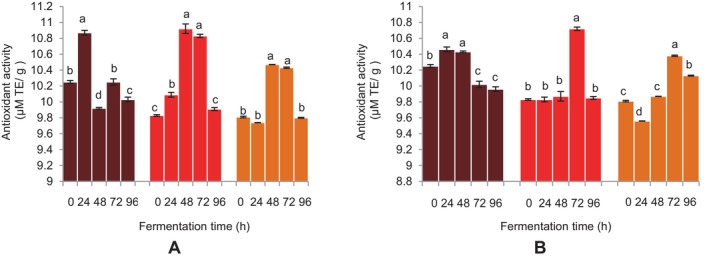
Effect of fermentation time on ABTS (2, 2′‐azinobis‐3‐ethylbenzthiazoline‐6‐sulfonic acid) radical scavenging activity of finger millet and sorghum malt beverages: 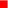 dark brown finger millet; 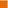 brown finger millet; and 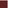 sorghum. (A) Fermentation with *Lactobacillus fermentum* . (B) Fermentation with grain microbial flora. Bars are mean values of triplicate determinations ± standard error. For each fermentation period, mean values with different letters are significant at *p* < 0.05.

**FIGURE 10:**
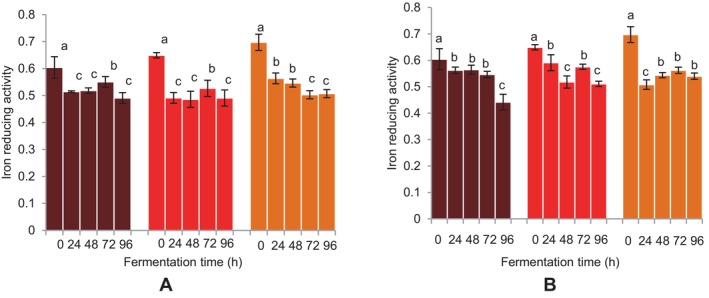
Effect of fermentation time on FRAP (ferric‐reducing antioxidant power) of finger millet and sorghum malt beverages: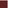 dark brown finger millet; 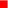 brown finger millet; 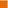 sorghum. (A) Fermentation with *Lactobacillus fermentum* . (B) Fermentation with grain microbial flora. Bars are mean values of triplicate determinations ± standard error. For each fermentation period, mean values with different letters are significant at *p* < 0.05.

Except the sorghum beverages, the ABTS^+^ radical scavenging activities of the FM beverages showed a substantial increase at 24 h of fermentation using both microbial sources (Figure 9A,B). At 48 h of fermentation, the BFM beverage's ABTS radical scavenging activity increased and continued to do so for the whole 72 h using *L. fermentum*. A similar effect was observed for sorghum beverages using *L. fermentum* . A decrease in the iron‐reducing activities of the beverages was seen at 24 h for both microbial sources (Figure 10A,B). An increase in iron‐reducing activities was also seen at 72 h for the FM beverages, which later reduced significantly at 96 h. A similar trend was noted for the sorghum beverage at 96 h of fermentation. Generally, fermentation for 96 h reduced the antioxidant activities of the FM and sorghum beverages, especially for *L. fermentum* .

## 4 Discussion

### 4.1 Effect of Fermentation Time on Viscosity, pH, and Sugar Content of FM and Sorghum Malt Beverages

The findings indicated that the FM beverages had a high viscosity that decreased as fermentation progressed. The viscosities of the FM beverages were of the normal flowing consistency of < 1 Pa.s., suitable for infant liquid foods (Mouquet and Tréche 2001). The observation can be linked to how microbial enzymes during fermentation break down complex carbohydrates.

Acid‐producing bacteria and other microorganisms that participate in lactic fermentation to form lactic acid and other metabolites from sugars and organic acids are associated with the elevated acidity of the beverages. A similar observation in pH or increased acidity in the fermentation of FM has been demonstrated by other researchers (Antony and Chandra 1997; Abioye et al. 2022; Joseph et al. 2024). The rise in sugar levels of the beverages is mostly linked to the enzymatic actions, mainly α‐amylases, of fermenting microbes that break starches like oligosaccharides through the cleavage of amylose and amylopectin into simple sugars as fermentation progresses. The sugar levels of the nonfermented beverages were below 0^o^brix. A similar observation was reported by Palmer (1992) and Zvauya, Mygochi, and Parawira (1997). Even though this finding has no obvious explanation, it could be inferred that there were no detectable sugars in the unfermented beverages. Since the increase in the sugar content of the fermented beverages was due to the enzymatic activity of the fermenting microbes, which hydrolyzed starches into simple sugars, the virtually nonexistent sugar content of the unfermented beverages could be a result of the fact that the samples had not undergone fermentation.

### 4.2 Effect of Fermentation Time on TP Content, TF Content, and Phenolic Compound Concentration of Fermented FM and Sorghum Malt Beverages

Few studies have examined the phenolic composition of fermented FM beverages; even fewer have reported the TP and TF levels. The study's extraction technique and the impact of fermentation on the concentration of phenolic compounds may be the cause of the low number of phenolics found. Thus, more research on extraction conditions for FM beverage phenolic profiling may be suggested by this data.

The fermentation process resulted in a decrease in the TPs, TFs, and phenolic components of the beverages. Hydrolysis, synthesis and metabolic utilization of phenolic compounds occur during fermentation (Bhat, Singh, and Sharma 1998; Kim et al. 2011; Hur et al. 2014). The ability of hydrolase enzymes such as glucosidase, phenolic acid reductase, and phenolic acid decarboxylase to metabolize phenolic acids and their esters and flavonoid glucosides during lactic fermentation has also been demonstrated to have a major influence on the polyphenol concentration and antioxidant activity of cereal grains, particularly red sorghum (Svensson et al. 2010; Udeh 2018; Jan et al. 2022). The decline in the phenolics with fermentation time could be attributed to their utilization resulting from the enzyme activities of the fermenting microorganisms during the fermentation process. The effect could also result from increased oxidation of the phenolic and flavonoid compounds due to the methanolic extraction and pasteurization temperatures used during the analysis and production of the beverages. The outcome aligns with previous reports, which have noted changes in the final products, such as a decrease in phenolic compounds as cereals ferment (Blandino et al. 2003; Towo, Matuschek, and Svanberg 2006; Kayode, Hounhouigan, and Nout 2007; Rani and Anthony 2014).

### 4.3 Effect of Fermentation Time on Citric Acid Content of FM and Sorghum Malt Beverages

Lactic acid bacteria ferment a variety of sugars and convert specific organic acids such as citric and malic acid into lactic acid, and depending on the strain, acetic acid, ethanol, and carbon dioxide are produced as well (De Vuyst and Weckx 2016; Udeh 2018). The bioconversion of citric acid by fermenting bacteria into acetic acids or other metabolites may account for the decline in citric acid levels in beverages during fermentation. Equally, citric acid is heat‐labile and can be denatured by heat treatment (Wyrzykowski et al. 2011; Udeh 2018). Thus, the reduction in the citric acid levels of the beverages might be partly linked to the influence of temperature on the citric acid during extraction and pasteurization of the beverages.

### 4.4 Effect of Fermentation Time on Antioxidant Activity of FM and Sorghum Malt Beverages

Fermentation plays a key role in antioxidant preservation owing to its modest temperature and restricted oxygen access, which ensures optimal conditions for decreased lipid oxidation (Udeh 2018). Moreover, research has established that the hydrolytic action of enzymes on conjugated phenolics, sugar residues, and esters of organic acids boosts the antioxidant potential of fermented foods (Grajek and Olejnik 2010). The antioxidant activities of the beverages could be linked to the additive action of individual phenolics as well as the release of esterified polymeric phenolics and organic acids with antioxidant effects.

The findings indicate that the type of substrate and length of fermentation had an impact on the beverages' antioxidant activity. Although no substantial change was noticed at 72 h, the FM beverages showed a fermentation‐time‐dependent decrease in the antioxidant activities, which were considerably reduced at 96 h of fermentation. Correia et al. (2004) noted a decline in antioxidant activity during the last stages of solid‐substrate fermentation of pineapple waste and soy flour composites using *Rhizopus oligosporus*. Additionally, Kim et al. (2011) observed that flavonol glycosides decreased during the fermentation of tea and that this decrease may have been caused by oxidative degradation. The research found that the tea catechins were reduced to aflavins and arubigin, which led to the loss of total soluble phenols and antioxidant action (Kim et al. 2011; Udeh 2018). This process could account for the decrease in antioxidant activities of beverages, particularly after 96 h of fermentation, where further disintegration of free and/or depolymerization of phenols occurred (Udeh 2018). A positive association is seen between the DPPH and FRAP radical scavenging activities of the beverages, as indicated by the correlation coefficients of their antioxidant activities (Table 3). The results provide further evidence of the fermentation‐time‐dependent decline in the antioxidant actions of the beverages using both microbial sources.

**TABLE 3:** Pearson correlation coefficients between DPPH, ABTS, and FRAP assays of fermented finger millet and sorghum malt beverages.

Response variables	Grain microbial flora			*Lactobacillus fermentum*		
	DPPH	ABTS	FRAP	DPPH	ABTS	FRAP
*Dark brown finger millet*						
DPPH	1.000	0.463	0.890**	1.000	0.234	0.718**
ABTS	0.463	1.000	0.452	0.234	1.000	0.195
FRAP	0.890**	0.452	1.000	0.718**	0.195	1.000
*Brown finger millet*						
DPPH	1.000	0.234	0.718**	1.000	0.234	0.718**
ABTS	0.234	1.000	0.195	0.234	1.000	0.195
FRAP	0.718**	0.195	1.000	0.718**	0.195	1.000
*Sorghum*						
DPPH	1.000	−0.242	0.744**	1.000	−0.275	0.914**
ABTS	−0.242	1.000	0.004	−0.275	1.000	−0.385
FRAP	0.744**	0.004	1.000	0.914**	−0.385	1.000

*Note:* Pearson correlation coefficients *, ** indicate significance at *p* < 0.05 and 0.01, respectively.

Abbreviations: ABTS, 2, 2′‐azinobis‐3‐ethylbenzthiazoline‐6‐sulfonic acid; DPPH, 2, 2‐diphenyl‐1‐picrylhydrazyl; FRAP, Ferric‐reducing antioxidant power.

## 5 Conclusion

The research suggests that FM malt can be utilized in the manufacture of nonalcoholic beverages with health benefits either with *L. fermentum* or grain microflora. The phenolic compounds established in the study are well associated with reduced risk of physiological diseases such as diabetes and hypertension. Moreover, catechin and epicatechin that were found in significant amounts in the FM beverages have been shown to exhibit anticancer and antiaging properties. Fermenting the FM malt flour for 24 h caused the retention of high levels of TP and had higher antioxidant activities compared to other fermentation durations. Fermenting the malt flour for 96 h negatively affected the antioxidant activities of the beverages. The FM beverages showed a measurable amount of citric acid that decreased with fermentation time. The pH and sugar levels of the FM beverages were optimal for the conservation of a typical nonalcoholic beverage. The FM beverage viscosities showed a drinkable texture, however, requires a matching sensorial evaluation. Further research on microbiological and sensory properties is needed to further validate the suitability of FM in the production of nonalcoholic beverages.

## Author Contributions

**Henry Okwudili Udeh:** conceptualization (lead), data curation (equal), formal analysis (lead), funding acquisition (equal), investigation (lead), methodology (lead), project administration (lead), resources (equal), validation (equal), writing – original draft (equal), writing – review and editing (equal). **Kwaku Gyebi Duodu:** conceptualization (equal), data curation (supporting), formal analysis (supporting), methodology (supporting), project administration (supporting), resources (supporting), supervision (equal), validation (supporting), writing – original draft (equal), writing – review and editing (equal). **Afam Israel Obiefuna Jideani:** conceptualization (equal), data curation (equal), formal analysis (equal), funding acquisition (lead), investigation (equal), methodology (equal), project administration (equal), resources (equal), software (equal), supervision (lead), validation (equal), visualization (equal), writing – original draft (equal), writing – review and editing (equal).

## Conflicts of Interest

The authors declare no conflicts of interest.

## Data Availability

The data presented in this study are available on request from the corresponding author.
